# WORKING WITH COMMUNITIES TO INCREASE DIVERSITY, EQUITY, AND INCLUSION IN GERONTOLOGICAL RESEARCH

**DOI:** 10.1093/geroni/igac059.635

**Published:** 2022-12-20

**Authors:** Stephanie Chow, Ronica Rooks

## Abstract

Gerontological researchers are encouraged to include older adults from underrepresented populations in their research and to think broadly about how they conceptualize diversity, equity, and inclusion (DEI) efforts in their work. This includes not only research on older adults from minority racial and ethnic communities, but also lesbian/gay/bisexual/trans*/queer+ (LGBTQ+) communities, as well as other cultural, religious, and socioeconomic groups. One of the most successful ways for reaching older adults in these populations is through community-based participatory research (CBPR). CBPR is a partnership approach to research that equitably involves community members, organizational representatives, researchers, and others in all aspects of the research process, with all partners in the process contributing expertise and sharing in the decision-making and ownership. Two papers in this symposium use CBPR to identify and include older adults from minority populations in their studies. A third paper reports on working with a Community Advisory Board to recruit bereaved dementia caregivers for research. The final paper walks us through steps to increase DEI efforts by meticulous research question development and thoughtful determinations regarding literature review terminology, recruitment efforts, data collection, and reporting and dissemination techniques.

